# The diagnostic benefit of 16S rDNA PCR examination of infective endocarditis heart valves: a cohort study of 146 surgical cases confirmed by histopathology

**DOI:** 10.1007/s00392-020-01678-x

**Published:** 2020-06-02

**Authors:** Christina Armstrong, Tim Christian Kuhn, Matthias Dufner, Philipp Ehlermann, Stefan Zimmermann, Christoph Lichtenstern, Jasmin Soethoff, Hugo A. Katus, Florian Leuschner, Alexandra Heininger

**Affiliations:** 1 Department of Internal Medicine III, Heidelberg University Hospital, Im Neuenheimer Feld 410, 69120 Heidelberg, Germany; 2 Division Hospital and Environmental Hygiene, Department of Infectious Diseases, Medical Microbiology and Hygiene, Heidelberg University Hospital, Heidelberg, Germany; 3 DZHK (German Centre for Cardiovascular Research), Partner Site, Heidelberg, Germany; 4 Division Bacteriology, Department of Infectious Diseases, Medical Microbiology and Hygiene, Heidelberg University Hospital, Heidelberg, Germany; 5 Department of Anesthesiology, Heidelberg University Hospital, Heidelberg, Germany; 6 Department of Cardiac Surgery, Heidelberg University Hospital, Heidelberg, Germany

**Keywords:** Infective endocarditis, PCR, Pathogen detection, Heart valve, Skin commensal

## Abstract

**Aims:**

Upon suspicion of infective endocarditis, the causative microorganism must be identified to optimize treatment. Blood cultures and culturing of removed valves are the mainstay of this diagnosis and should be complemented by growth-independent methods. We assessed the diagnostic benefit of examining removed endocarditis valves by broad-range bacterial PCR to detect causative bacteria in cases where culturing was not available, negative, or inconclusive because a skin commensal was detected, in patients from our clinical routine practice.

**Methods and results:**

Patients from Heidelberg University Hospital with suspicion of endocarditis, followed by valve replacement and analysis by 16S rDNA PCR, between 2015 and 2018, were evaluated. 146 patients with definite infective endocarditis, confirmed by the valve macroscopics and/or histology, were included. Valve PCRs were compared to corresponding blood and valve culture results. Overall, valve PCR yielded an additional diagnostic benefit in 34 of 146 cases (23%) and was found to be more sensitive than valve culture. In 19 of 38 patients with both negative blood and valve cultures, valve PCR was the only method rendering a pathogen. In 23 patients with positive blood cultures detecting skin commensals, 4 patients showed discordant valve PCR results, detecting a more plausible pathogen, and in 11 of 23 cases, valve PCR confirmed commensals in blood culture as true pathogens. Only the remaining 8 patients had negative valve PCRs.

**Conclusion:**

Valve PCR was found to be a valuable diagnostic tool in surgical endocarditis cases with negative blood cultures or positive blood cultures of unknown significance.

**Trial registration:**

S-440/2017 on 28.08.2017 retrospectively registered.

**Graphic abstract:**

Subdividing of all infective endocarditis patients in this study, showing that valve PCR yields valuable information for patients with skin commensals in blood cultures, which were either confirmed by the same detection in valve PCR or refuted by the detection of a different and typical pathogen in valve PCR. Additionally, benefit was determined in patients with negative or not available blood cultures and only positive detection in valve PCR. +: Positive; −: negative; n/a: not available results

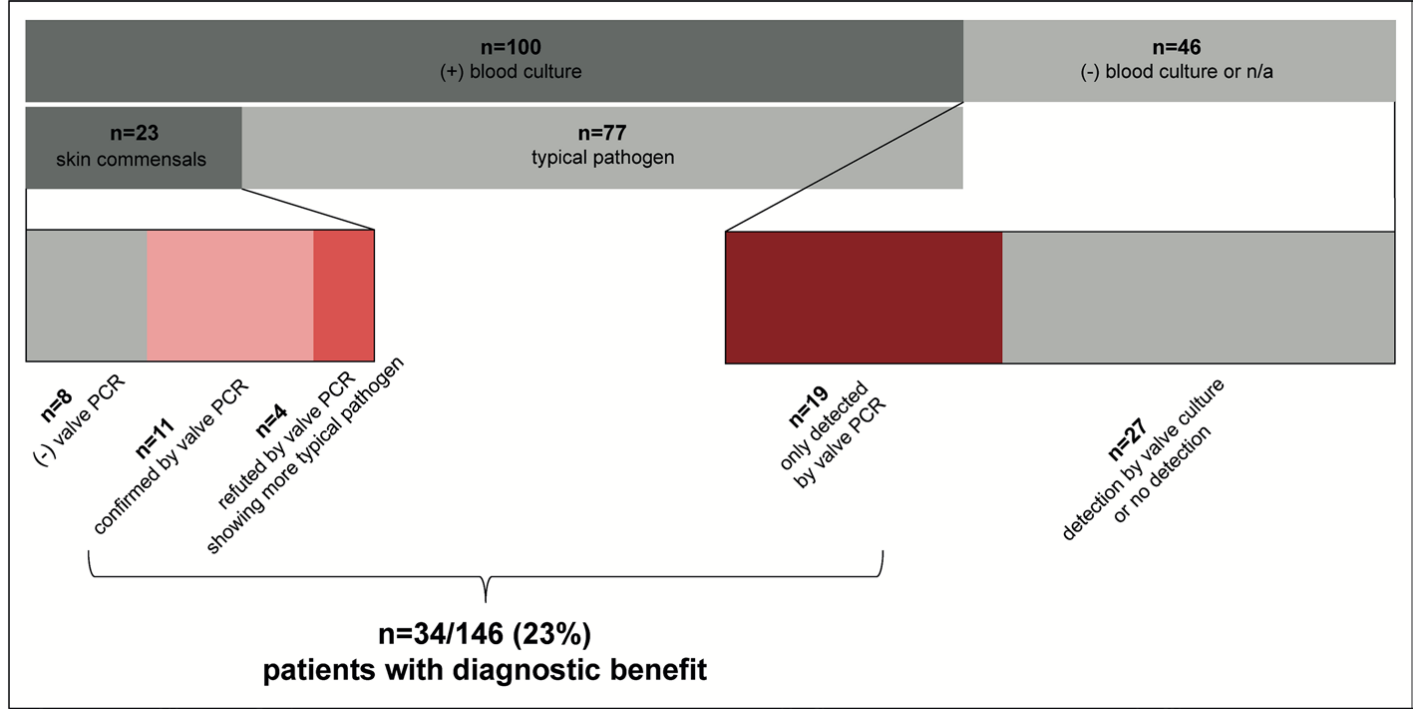

**Electronic supplementary material:**

The online version of this article (10.1007/s00392-020-01678-x) contains supplementary material, which is available to authorized users.

## Introduction

Infective endocarditis remains a clinical challenge, with often complicated courses, up to 50% of endocarditis patients needing surgical valve replacement, and an in-hospital mortality of up to 30% [1–3]. Identification of the causative microorganism (pathogen) and if possible, antibiotic drug susceptibility testing, are prerequisites for optimal antimicrobial therapy, and are currently done using blood cultures and culturing of removed valves. Should these remain negative, guidelines recommend alternative methods to achieve pathogen detection in as many patients as possible [1, 4, 5].

While most blood cultures should be positive [6, 7], clinical experience suggests that about one-third of cases remain blood culture-negative [1]. Cases of blood culture-negative endocarditis are most often caused by prior antibiosis inhibiting growth in blood samples, but can also be caused by intracellular (e.g. *Coxiella burnetii*), fastidious, slow-growing or unculturable microorganisms (e.g. *Tropheryma whipplei*), as well as incorrect sampling or processing, or non-infective endocarditises [6, 8, 9].

The microbiological diagnosis of blood culture-negative endocarditises before surgery should be assisted by serology and specific PCR analysis of the blood [1]. Fournier et al. describe specific PCR of the blood being positive for patients with *Coxiella burnetii* in 53% of cases, in which detection could also be achieved by specific valve PCR, whereas 16S rDNA (broad-range) PCR of the blood only achieved a sensitivity of 14% [8]. For *Tropheryma whipplei*, specific PCR of the valve can achieve detections in up to 100% of cases [10]. Specific PCR, however, is not suitable to screen for a spectrum of pathogens, and 16S rDNA PCR of the blood not sensitive enough so that many hospitals do not perform blood PCR in routine diagnostics.

In the case of surgery, it is more promising to examine the removed valve by culture, immunohistochemistry, and 16S rDNA PCR [1].

Although valve culture is still the gold standard for all surgical infective endocarditises, it often remains negative due to the aforementioned limitations of culturing [8, 11]. Therefore, valve PCR has been established as a growth-independent and rapid method to assist pathogen detection [12].

For blood culture-negative endocarditises, 16S rDNA PCR of the valve has an accepted sensitivity of around 66% and its diagnostic benefit has been established [8]. For blood culture positive endocarditises, valve PCR can confirm previously detected pathogens or reject microorganisms detected in prior blood cultures for other probable pathogens [13]. The British Society for Antimicrobial Chemotherapy considers 16S rDNA valve PCR a Minor Duke Criterion [5], whereas the European Society of Cardiology recommends specific blood and valve PCR for blood culture-negative endocarditises [1]. In contrast, the American Heart Association only acknowledges a benefit from valve PCRs without further specification [4].

Heidelberg University Hospital has implemented the 16S rDNA PCR of removed valves as a secondary method for patients with infective endocarditis suspected before or during surgery. We examined if 16S rDNA valve PCR can improve the diagnostic yield in patients with endocarditis confirmed during surgery and/or by histology in cases where skin commensals appeared in blood cultures and cases where blood cultures were negative or not available.

## Methods

### Patients

All adult patients in the cardiothoracic surgery department of Heidelberg University Hospital, with surgical valve replacement under the suspicion of IE, from January 2015 to December 2018, were evaluated. Suspicion was based on clinical, microbiological, echocardiographic and imaging findings before surgery [1, 14], or the description of endocarditis formations during surgery. Within this group, those patients with definite infective endocarditis according to the Pathologic Duke Criteria and 16S rDNA PCR examination of their valves were included.

### Valves and associated valve material

Native and prosthetic heart valves, valved conduits, and vegetations were analyzed in this study. Results from surgical swabs and infections on intracardiac devices were excluded.

### Valve processing

Immediately after surgery, the valve was halved under sterile conditions. One fragment was sent for histological evaluation and the other for microbiological examination by culture and PCR. For the latter, the fragment was halved a second time. Tissue from prosthetic valves and vegetations could not be halved so that surgeons took samples from visually suspicious areas of the valve for microbiological diagnostics.

### Confirmation of IE

#### Macroscopic evaluation

During surgery, the macroscopics of the valve in situ and immediately after removal were evaluated by the surgeon in accordance with the Pathological Duke Criteria (vegetations and intracardiac abscess) [15]. Paravalvular leakage was not included, as it is also a characteristic of heart valve disease [16].

#### Histological evaluation

Histological analysis was performed according to the Pathological Duke Criteria and routine standards [15]. Hereby an active infective endocarditis was defined as a florid, fibrinous, ulcerous, ulceropolypous and polypus infective endocarditis. If eosinophilic infiltration, chronic inflammation or past endocarditises were found on removed valves, these were not considered.

### Microbiological methods

#### Preoperative blood cultures

Results of blood cultures within 6 months before surgery, found in inhouse documentation and records from referring hospitals, were used. Blood cultures have been taken according to guidelines [1], although not all patients had at least three blood cultures taken or more than two positive blood cultures confirming a microorganism, because in some cases endocarditis was only suspected intraoperatively. Processing of blood cultures, identification of isolates and drug susceptibility testing were performed according to the German Microbiological-Infectious Quality (MiQ) Standards [17]. If different microorganisms were detected in the same patient, these were all represented in the results.

#### PCR of valve

16S rDNA PCR proceeded according to microbiological routine standards and was performed with the eubacterial primers 357f (5′-CCTACGGGAGGCAGCAG-3′) and 519r (5′-ATTACCGCGGCK¬GCTGG-3′) [18, 19]. If indicated, organism-specific PCRs could be performed with targeted primers; such data was not included in our analysis. Detected microorganisms were specified through the sequencing of the amplification product and comparison to the BIBI database (https://umr5558-sud-str1.univ-lyon1.fr/lebibi/lebibi.cgi) [20].

#### Culture of valve

Valve culture proceeded according to the Duke Criteria and microbiological routine standards [15]. Columbia, chocolate, and MacConkey agars were incubated at 37 °C in 5% CO_2_ for 24 to 48 h. Schaedler and neomycin-vancomycin agars were incubated at 37 °C in an anaerobic chamber (GasPak; Becton, Dickinson, Franklin Lakes, NJ) for 48 h. Chromogenic *Candida* agar was incubated at 37 °C in 5% CO_2_ for 24 to 48 h. Plates were reviewed after 24 and 48 h. Samples with no growth were further incubated under the same conditions for 120 h. Colonies were identified by matrix-assisted laser desorption ionization–time of flight (MALDI-TOF) (Bruker Daltonics, Billerica, USA). Antibiotic susceptibility was determined routinely by Vitek 2 (bio-Mérieux, Marcy l’Étoile, France) using the European Committee on Antimicrobial Susceptibility Testing (EUCAST) guidelines for interpretation. If different microorganisms were detected on the same valve, these were all represented in the results.

#### Serological examination

Considering guidelines, serology was performed for blood culture-negative endocarditises and if patient characteristics indicated so [1].

#### Definitions

Negative results had no growth of microorganisms. Some results were not available due to inconsistent clinical management, incomplete transfer of referral documents, rapid disease progression, so that documents were not yet available at the time of surgery and cases in which infective endocarditis was only suspected during surgery so that preoperative blood cultures had not proceeded. Valve culture and PCR were the only microbiological diagnostics for these patients.

#### Data processing

Data were available on the IS-H/i.s.h.med.^®^ (SAP, Walldorf, Germany) and GSM/ARCHIV (AGFA Healthcare, Stuttgart, Germany) servers. It was collected in Microsoft Excel (Microsoft Corp, Redmond, WA, USA). Analysis was done in Microsoft Excel and in R (R Foundation for Statistical Computing, Vienna, Austria). Figures and tables were created in Microsoft Word and Excel.

### Statistical analysis

Statistical differences between the positivity of valve culture versus PCR, and between the diagnostic benefit of valve culture versus PCR, were tested for using McNemar’s chi-squared test. Four patients with not available valve cultures were excluded from this analysis (Table S1).

## Results

This retrospective observational cohort study examined the diagnostic value of valve PCR, considering corresponding blood and valve cultures. We evaluated (1) how many and which microorganisms were detected by blood culture preoperatively, and valve culture and PCR postoperatively, (2) in which patients clinically concordant species were detected by different methods, and (3) if there was a diagnostic benefit from valve PCR.

### Patients

Of all patients with valve removal from January 2015 to December 2018 at our hospital, 151 patients had a suspected infective endocarditis before or during surgery and their valves examined by PCR. In 5 of these patients infective endocarditis was not proven, while in 146/151 patients infective endocarditis was confirmed by the histopathology of the valve. These 146 patients were further examined and their characteristics are displayed in Table 1.

**Table 1:** Characteristics of endocarditis patients and valves replaced

Characteristics	*n* (%)
*n* = 146 patients with confirmed endocarditis	
Age mean ± SD	62 ± 12
Male sex	109 (75)
Single valve replaced	112 (77)
Multiple valves replaced^a^	34 (23)
*n* = 182 valves replaced	
Native valves	159 (87)
Aortic	73 (46)
Mitral	74 (46)
Tricuspid	11 (7)
Pulmonal	1 (1)
Prosthetic valves	23 (13)
Mechanical	11 (48)
Bioprosthetic	11 (48)
Conduit	1 (4)

^a^Two patients had their aortic, mitral and tricuspid valves replaced simultaneously

### Positivity of microbiological methods

**Fig. 1:**
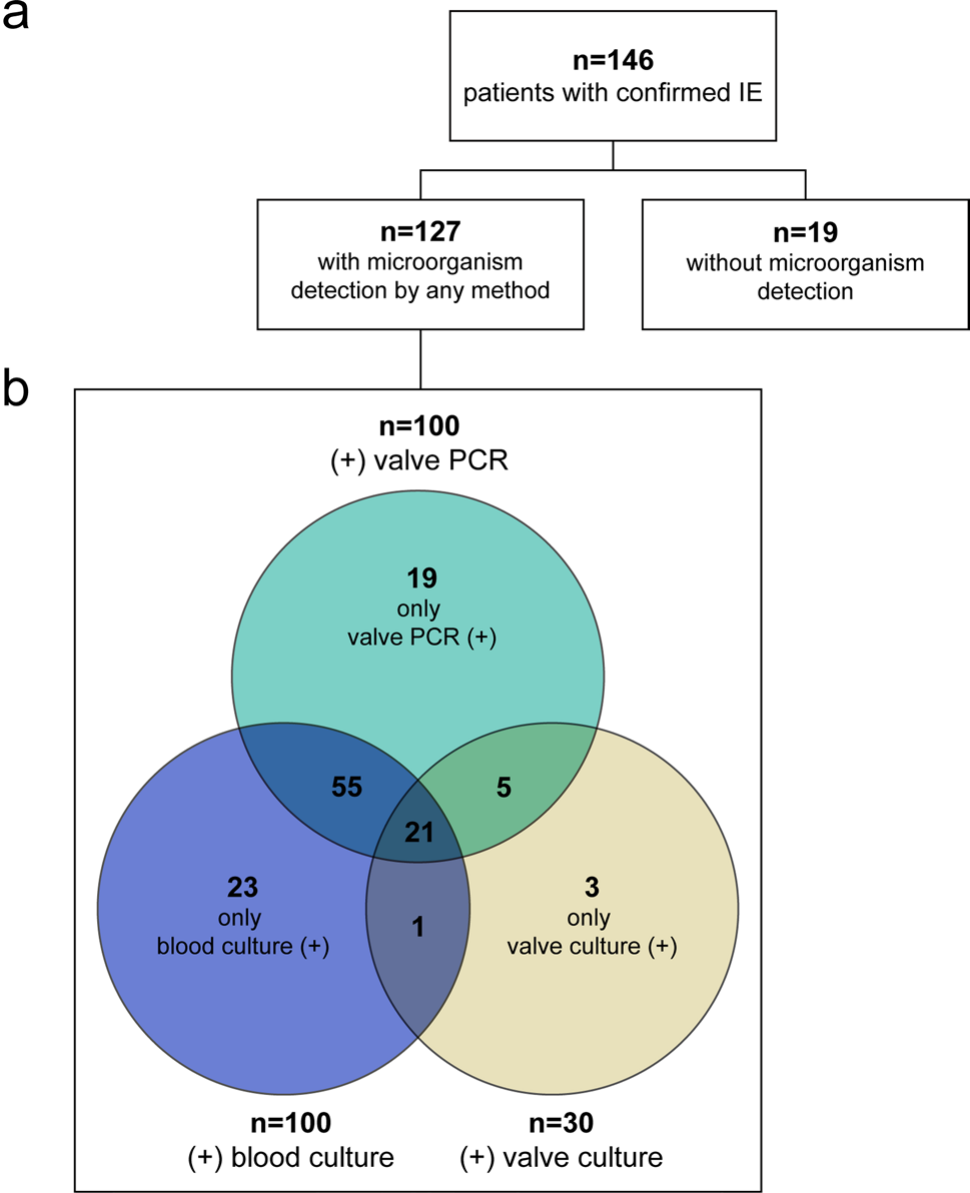
**a** Flowchart of infective endocarditis (IE) patients with microorganism detection. **b** Venn diagram comparing detection by each microbiological method. +: Positive; −: negative results

In 127/146 patients a microorganism was detected. In 131 patients blood cultures, in 142 valve cultures and in 146 valve PCRs were available. In 100/131 (76%), 30/142 (21%) and 100/146 (68%) patients, microorganisms were detected by blood cultures, valve cultures and valve PCRs, respectively (Fig. 1). Detection groups overlapped, as patients had positive detections in multiple methods. Valve PCR detected microorganisms significantly more often than valve culture (97/142 vs. 30/142) (*X*^2^ = 58.08, *p* < 0.001). 19/146 patients remained without microbiological detection, despite applying at least two, if not all of these methods.

### Microorganism distribution

In 127 patients with microorganism detections a total of 147 microorganisms were found by any of the three microbiological methods. The total number of microorganisms exceeds the number of detections because 7 blood and 4 valve cultures found two microorganisms in each culture (Table S2), 9 patients had different species between methods (Table S3) and 1 patient had a valve PCR detection of eubacterial DNA, which had to be counted separately.

The distribution of microorganisms detected is shown in Table 2, whereby no differentiation between cases in which concordant or different microorganisms were detected in a single patient was made. *Streptococcus spp.*, *Staphylococcus aureus*, and coagulase-negative staphylococci (CoNS) were the main pathogens detected. Among the other microorganisms, 16S rDNA valve PCR detected 2 *Coxiella burnetii* and 2 *Tropheryma whipplei*, which were confirmed by subsequent serology and specific PCR of the valves respectively.

**Table 2:** Distribution of microorganisms detected by each microbiological method

Species	Number of microorganisms detected by each method *n* (%)			
	*n* = 147^a^	*n* = 107	*n* = 100	*n* = 34
	All methods	By blood culture	By valve PCR	By valve culture
*Streptococcus* spp.	51 (35)	38 (36)	45 (45)	5 (15)
*Staphylococcus aureus*	25 (17)	24 (22)	16 (16)	7 (21)
CoNS	33 (22)	25 (23)	15 (15)	11 (32)
*Enterococcus* spp.	11 (7)	9 (8)	10 (10)	4 (12)
Others	27 (18)	11 (10)	14 (14)	7 (21)

^a^127 patients had detections with a total of 147 microorganisms identified, because different microorganisms between methods and multiple microorganisms in a single method were detected

The microorganism distributions between methods differ, because exposure to preoperative antibiosis varied, seeing that blood cultures had proceeded earlier than valve culture and PCR. More information on the other microorganisms detected and which microorganisms were detected by the individual methods can be found in List S1.

### Concordance of valve PCR with preoperative blood cultures

**Fig. 2:**
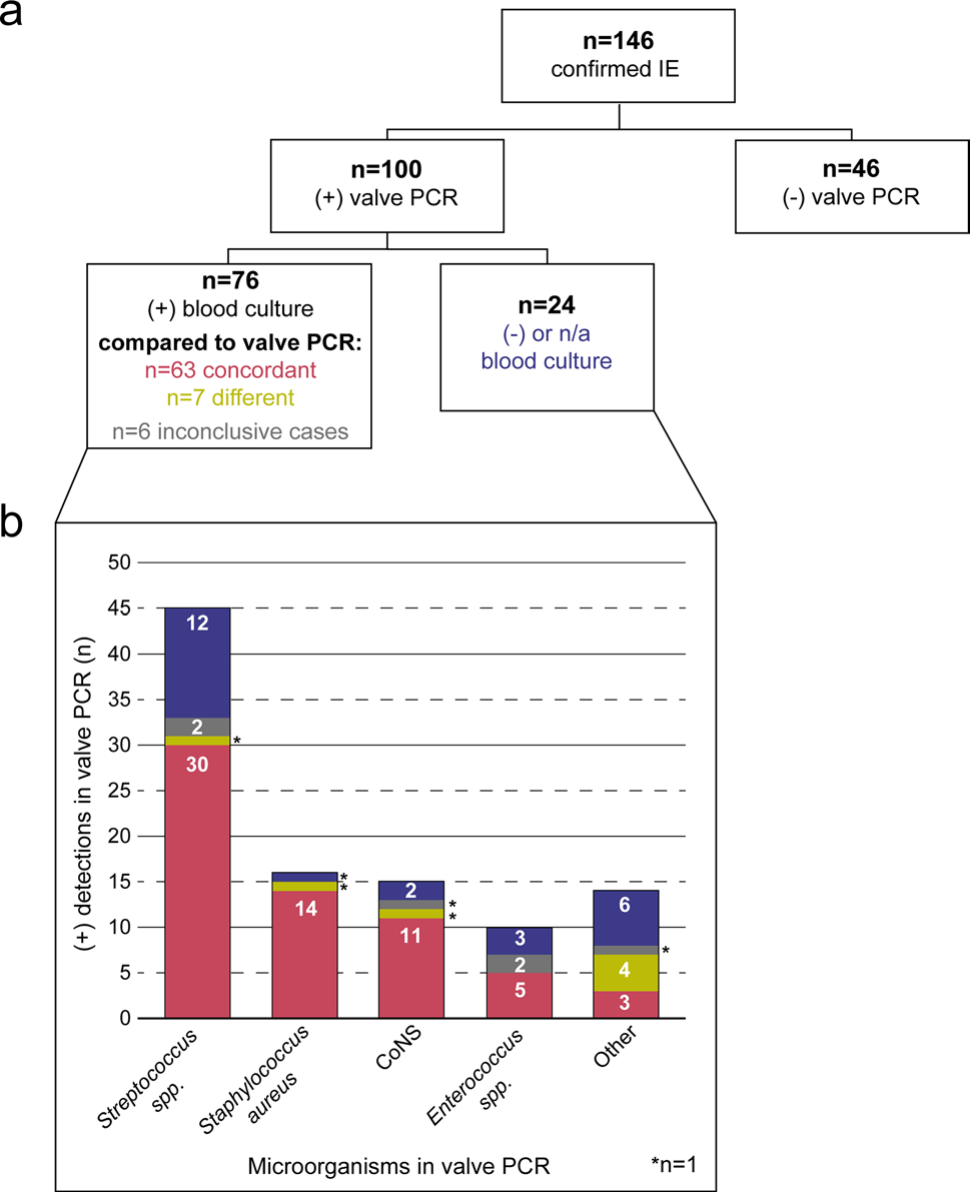
**a** Flowchart of microorganism detections by valve PCR and blood cultures. **b** Distribution of microorganisms detected by valve PCR; blood cultures are concordant or different compared to valve PCR or inconclusive, negative or not available. +: Positive; −: negative; n/a: not available results

**Fig. 3:**
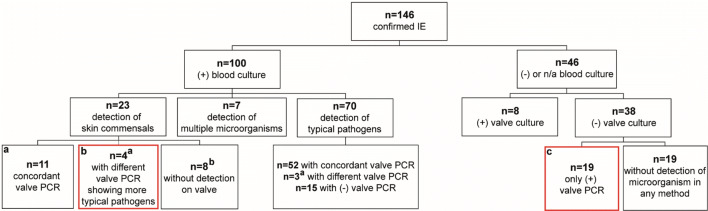
Flowchart of infective endocarditis (IE) patients with diagnostic benefit from valve PCR, shown by red box and detailed in Table 3 under the corresponding letters. +: Positive; −: negative; n/a: not available results; ^a^marks patients with the difference between blood culture and valve PCR, also in Table S2; ^b^marks 7 patients with negative valve PCRs and 1 case with eubacterial DNA in PCR, but unsuccessful species identification; in all 8 patients valve culture remained negative

Of 100/146 patients with positive valve PCRs 76/100 also had earlier positive blood cultures. For 63/76 patients concordant species were found in blood cultures and valve PCR. In 7/76 patients blood cultures and valve PCR differed regarding the species detected (Fig. 2a, b, Table S2). 4 of these 7 patients had skin commensals in blood cultures but a more plausible causative microorganism in valve PCR (Fig. 3 and Table 3b), whereas in 3/7 patients a typical pathogen for infective endocarditis was detected in blood cultures, but DNA of a different species in valve PCR. In 6/76 patients a meaningful comparison of findings of blood culture versus valve PCR could not be done (inconclusive cases). In 1/6 of these PCR detected eubacterial DNA, but sequencing was not able to identify a species and in 5/6 inconclusive cases multiple microorganisms were isolated in blood culture but only a single microorganism was found by the respective valve PCRs (Table S3).

**Table 3:** Diagnostic benefit of valve PCR for patients

	Blood culture	Valve PCR	Valve culture
(a) Patients with coagulase-negative staphylococci in blood cultures that are confirmed by concordant valve PCR			
1	*Staphylococcus cohnii*	*Staphylococcus capitis*	*Staphylococcus capitis*
2	*Staphylococcus epidermidis*	*Staphylococcus epidermidis*	−
3	*Staphylococcus epidermidis*	*Staphylococcus epidermidis*	−
4	*Staphylococcus epidermidis*	*Staphylococcus epidermidis*	*Staphylococcus epidermidis*
5	*Staphylococcus epidermidis*	*Staphylococcus epidermidis*	*Staphylococcus epidermidis*
6	*Staphylococcus epidermidis*	*Staphylococcus epidermidis*	*Staphylococcus epidermidis*
7	*Staphylococcus epidermidis*	*Staphylococcus epidermidis*	*Staphylococcus epidermidis*
8	*Staphylococcus haemolyticus*	*Staphylococcus epidermidis, Staphylococcus haemolyticus*	−
9	*Staphylococcus haemolyticus*	*Staphylococcus spp.*	−
10	*Staphylococcus lugdunensis*	*Staphylococcus lugdunensis*	−
11	*Staphylococcus warneri*	*Staphylococcus warneri*	−
(b) Patients with skin commensals in blood cultures, but typical pathogen for endocarditis in valve PCR			
1	*Corynebacterium jeikeium*	*Tropheryma whipplei*	−
2	*Staphylococcus epidermidis*	*Coxiella burnetii*	−
3	*Staphylococcus epidermidis*	*Staphylococcus aureus*	−
4	*Staphylococcus spp.*	*Streptococcus mitis/oralis*	−
(c) Patients with only positive valve PCR			
1	n/a	*Abiotrophia defectiva*	−
2	−	*Aggregatibacter actinomycetemcomitans*	−
3	n/a	*Corynebacterium kroppenstedtii*	−
4	−	*Coxiella burnetii*	−
5	−	*Enterococcus faecalis*	−
6	−	*Klebsiella oxytoca*	−
7	−	*Staphylococcus epidermidis*	−
8	−	*Staphylococcus haemolyticus*	−
9	−	*Streptococcus agalactiae*	−
10	−	*Streptococcus dysgalactiae*	−
11	−	*Streptococcus gordonii*	−
12	−	*Streptococcus milleri*	−
13	n/a	*Streptococcus mitis*	−
14	−	*Streptococcus mitis*	−
15	−	*Streptococcus mitis*	−
16	−	*Streptococcus mitis*	−
17	−	*Streptococcus pasteurianus*	−
18	−	*Streptococcus salivarius*	−
19	−	*Tropheryma whipplei*	−

−: Negative; n/a: not available results

Of 100/146 patients with positive valve PCRs 24/100 patients had no pathogen detection in blood cultures (Fig. 2a, b), and 19/24 patients had no detections in their valve culture either (Fig. 3 and Table 3c). That means for 19/100 patients with negative or not available blood cultures, valve PCR was the only method of detection, identifying species such as *Streptococcus spp.*, *Coxiella burnetii* and *Tropheryma whipplei* (Table 3c). In the remaining 5/24 patients a microorganism was isolated by valve culture, which was concordant to that detected by valve PCR. Of 46/146 patients with negative valve PCRs 27/46 patients had a positive blood or valve cultures, and in only 19/46 patients were no microorganisms detected by any method.

### Concordance of valve PCR with valve culture

26/100 patients with positive valve PCRs also had a positive valve culture, and 19/26 had concordant microorganisms in their valve PCR and culture.

In 3/26 patients the species identified by valve PCR and culture were different (Table S2), and in 4/26 patients valve PCR identified a single microorganism, whereas valve culture detected multiple microorganisms (Table S3).

Concordant results in all three methods occurred in only 17/146 patients, mainly due to the poor sensitivity of valve culture.

### Diagnostic benefit of valve PCR

For 34/146 (23%) patients valve PCR yielded valuable information by detecting a microorganism when earlier blood cultures had detected a skin commensal, or were negative or not available. Valve PCR was significantly more likely to be of such diagnostic benefit than valve culture (32/142 vs. 8/142) (*X*^2^ = 17.63, *p* < 0.001).

Of 131 patients with blood culture results available 100 patients had positive blood cultures, and in 23/100 patients, the isolate detected was a skin commensal (CoNS*, Corynebacterium spp.* and *Propionibacterium acnes*). Diagnostic benefit was seen for 11/23 patients, in which the skin commensal detected in blood culture was also detected by valve PCR (Fig. 3 and Table 3a); that means an isolate of unclear dignity could be confirmed as the true pathogen. Of these patients 3 had had detection in a single blood culture and 8 in multiple blood cultures. Valve culture detected the concordant microorganism for 5 of these patients as well. In 4/23 cases with skin commensals in a single blood culture diagnostic benefit was also obtained by valve PCR, when DNA of a different bacterial species, considered a more typical pathogen of endocarditis, was identified (Fig. 3 and Table 3b); valve culture remained negative in these cases. Additionally, in 46/146 patients with negative or not available blood cultures 19/46 patients had their only positive detection in valve PCR, which for these cases was of diagnostic benefit as well (Fig. 3 and Table 3c).

## Discussion

This study evaluated the benefit of valve PCR for pathogen identification in real-life surgical patients with confirmed infective endocarditis. We describe a new subgroup of patients who gained valuable information from an examination of the valve by 16S rDNA PCR, namely those with skin commensals in blood cultures. Our results also confirm observations by Fournier et al. and Goldenberg et al. that blood culture-negative endocarditises benefit from detection through valve PCR and that blood culture positive infective endocarditises can be substantiated by concordant valve PCRs [8, 13]. Valve PCR was also significantly more successful in pathogen identification than valve culture, a tendency that has already been described [21, 22].

Our observed 46/146 (32%) surgical endocarditises with negative or not available blood cultures agree with the prevalence of blood culture-negative endocarditises described in guidelines [1]. Within this group we found valve PCR to be the only positive method of detection in 19/46 patients. Most blood culture-negative endocarditises had *Streptococcus *spp*.* identified by valve PCR, as would be expected based on the results of other established work [8]. This occurrs because many patients receive antibiosis before blood sampling, which causes blood cultures to remain negative even in cases with typical and easily culturable pathogens [23]. In addition, we found 2 patients with *Coxiella burnetii* and 2 with *Tropheryma whipplei* in their PCRs, which also agrees with existing results [8].

The cases with *Tropheryma whipplei* underline the benefit of valve PCR in the presence of fastidious microorganisms [10], as neither blood culture nor valve culture reliably achieve detection. This is unsettling considering that *Tropheryma whipplei* is known to cause at least 1%, and depending on regional differences substantially more, cases of infective endocarditis [8].

In this study a total of 34/146 (23%) patients with surgical infective endocarditis benefitted from 16S rDNA valve PCR, when PCR allowed skin commensals previously isolated by blood cultures to be reclassified as contaminants or true pathogens, or when PCR was the only method of detection. Peeters et al. described a diagnostic benefit of 16S rDNA valve PCR in 17% of surgical cases with confirmed infective endocarditis. These cases either had negative blood cultures beforehand or had a more plausible pathogen in valve PCR than had been previously detected in blood cultures [22]. Halavaara et al. also described a benefit of valve PCR for 14% of surgical blood culture-negative endocarditises [21], while valve culture was of no diagnostic benefit. This agrees with our results, where valve PCR was significantly (*p* < 0.001) more likely to provide clinically relevant information than valve culture.

In addition, valve PCR can substantiate earlier results of blood culture positive infective endocarditises [13]. Halavaara et al. describe 62% of surgical patients with such confirmed results [21], while we found concordant findings of blood cultures and valve PCRs in 63/146 (43%) of surgical infective endocarditises. The majority of cases with different microorganisms detected by blood culture and valve PCR in our study occurred when skin commensals appeared in blood culture but were refuted by valve PCR, which identified a typical pathogen on the valve (Table S3); for these patients valve PCR provided a diagnostic benefit.

To our knowledge, we are the first group to emphasize this benefit of 16S rDNA valve PCR for infective endocarditis patients with skin commensals in previous blood cultures. In clinical practice, skin commensals that are repeatedly isolated in blood cultures are considered causative [15, 24]. We were able to confirm that the majority of patients with repetitive findings of skin commensals in blood cultures, had concordant species on their valve, detected by a culture-independent method, namely valve PCR.

Such confirmation allowed antibiosis to be safely tailored to the species and unnecessary broad antibiosis, which frequently results from diagnostic uncertainty [25], to be avoided. In contrast in 4/23 patients 16S rDNA PCR identified a species typically causing infective endocarditis and thereby indicated that the previous isolation of skin commensals in the blood had been misleading. In these cases, PCR was essential to initiate an effective antibiosis against pathogens such as *Coxiella burnetii* or *Tropheryma whipplei*, which are not covered even by the broad empirical antibiotic regimes recommended for blood culture-negative endocarditis in guidelines [4]. These patients illustrate the value of 16S rDNA valve PCR to reduce diagnostic uncertainties and the risk of antibiotic overtreatment on the one hand, or insufficient antibiotic therapy on the other hand.

Even though PCR is a growth-independent method and less susceptible to antibiosis than culturing, the yield of PCR differs depending on the duration of effective antibiotic treatment that has been applied before the examination of valve material. Halavaara et al. describe 16S rDNA valve PCR being positive in 91% and valve culture in 41% of endocarditis patients receiving < 2 weeks of preoperative antibiosis; in all patients with ≥ 2 weeks of treatment only 53% of PCRs were positive and valve culture remained negative altogether [21]. Vollmer et al. also proposes that valve PCR is the more suitable method after prolonged antibiosis, even if the yield is reduced [12].

For this reason, and because PCR only requires a small amount of DNA for successful detection, Peeters et al. and Liesman et al. endorse prioritizing valve PCR over valve culture to maximize diagnostic yield [22, 26]. These findings correspond with our results, where the majority of patients received prolonged antibiosis before complications led to surgery, and still valve PCR was significantly (*p* < 0.001) more likely to be positive than valve culture.

The sensitivities of blood cultures cannot be compared with those of valvular methods, because blood cultures are taken much earlier before surgery, and patients will always have received more antibiosis before valvular examination [27].

Besides remaining negative, blood and valve cultures also cause confusion if multiple microorganisms are detected, and it is unclear if none, one or both are the true pathogens or contaminants. We detected 11/146 such cases (Table S2), of which 8 patients had multiple microorganisms in cultures, but a single detection in valve PCR. For these cases, it remains to be determined if PCR can reliably identify all pathogens in a single sample [25].

Blood and valve cultures are still the only methods in which antimicrobial drug susceptibility can be routinely performed. This is important given that resistances are on the rise and affect the main pathogens of infective endocarditis, namely *Streptococcus *spp*.* and *Staphylococcus aureus* [23, 28]. Susceptibility testing is based on the microbiological isolation of pathogens and therefore prone to the limitations of culturing; complete processing of samples usually requires 24 to 72 h [25]. Thus, growth-independent and quicker methods for antibiotic susceptibility testing are urgently needed and being intensely explored [29]. However, presently and because other methods have limitations as well, culture-based diagnosis still remains the gold standard [1].

To analyze removed valves by multiple methods, segmentation of the material is necessary. As infective tissue might not be distributed equally on the valve, this could impact subsequent analysis [12].

Recommendations on standardized valve partitioning might help to minimize the false-negative results due to this problem. Because valve PCR can detect very small amounts of DNA, it is the least susceptible to this error. However, valve PCR can also deliver unclear positive results when microorganism residues persist on valves, even though a full antibiosis cycle has been completed and clinical remission has been assumed [30, 31].

Contamination may also cause false-positive results; whereby the use of routine standards has minimized such errors [32]. This study is limited by its retrospective observational nature and single-center design. Patient selection was restricted to surgical cases with pathologically definite infective endocarditises, who received valve PCR at our hospital. Possible or rejected infective endocarditises and those with conservative treatment were not evaluated. Also, stratification according to patient characteristics and comorbidities did not proceed, even though these are known to impact the development of endocarditis [33, 34]. Methods such as serology and specific valve PCR were not routinely included, because these are only applied when a specific pathogen is already suspected. Sometimes diagnostics did not proceed as recommended, representing the real-life challenges faced in clinical practice [22]. Direct comparison of our results to those in literature must consider differences in study design, patient inclusion criteria and disease characteristics. Our conclusions could also be improved by examination in a prospective design.

## Conclusion

Considering challenges and limitations that remain when diagnosing endocarditis, 16S rDNA PCR of removed valves should complement existing diagnostics to increase the number of successful pathogen detections. In our cohort, patients with skin commensals in blood cultures proved to be a subgroup that benefits from valve PCR, in addition to those cases with negative or not available blood cultures. 16S rDNA PCR is an easy method that can make a significant difference for these endocarditis patients and helps to tailor antibiotic therapy.

## Electronic supplementary material

Below is the link to the electronic supplementary material.

Additional results on the patients not included in statistical analysis, a detailed distribution of the microorganisms detected in each of the methods, and the microorganisms detected in the different methods for patients with multiple detections in a single method and different species between methods, are detailed in this section. (DOCX 54 kb)

## Data Availability

Raw data is available for further analysis; please contact corresponding authors.
