# Supplementary material for: The diagnostic benefit of 16S rDNA PCR examination of infective endocarditis heart valves: a cohort study of 146 surgical cases confirmed by histopathology
Source: Clin Res Cardiol. 2020 Jun 2;110(3):332–42. doi: 10.1007/s00392-020-01678-x (PMC7906935; doi:10.1007/s00392-020-01678-x)
Supplement: Supplementary file 1 — Additional results on the patients not included in statistical analysis, a detailed distribution of the microorganisms detected in each of the methods, and the microorganisms detected in the different methods for patients with multiple detections in a single method and different species between methods, are detailed in this section. (DOCX 54 kb) [file 392_2020_1678_MOESM1_ESM.docx]

**Supplementary Material**

**The diagnostic benefit of** **16S rDNA PCR examination of infective endocarditis heart valves: a cohort study of 146 surgical cases confirmed by histopathology.**

in Clinical Research in Cardiology

Christina Armstrong^1,2^*, Tim Christian Kuhn^1,3^*, Matthias Dufner^1^, Philipp Ehlermann^1^, Stefan Zimmermann^4^, Christoph Lichtenstern^5^, Jasmin Soethoff^6^, Hugo A. Katus^1,3^, Florian Leuschner^1,3§^, Alexandra Heininger^2§^

1 Department of Internal Medicine III, Heidelberg University Hospital, Heidelberg, Germany

2 Department of Infectious Diseases, Medical Microbiology and Hygiene, Division Hospital and Environmental Hygiene, Heidelberg University Hospital, Heidelberg, Germany

3 DZHK (German Centre for Cardiovascular Research), Partner Site Heidelberg, Germany

4 Department of Infectious Diseases, Medical Microbiology and Hygiene, Division Bacteriology, Heidelberg University Hospital, Heidelberg, Germany

5 Department of Anesthesiology, Heidelberg University Hospital, Heidelberg, Germany

6 Department of Cardiac Surgery, Heidelberg University Hospital, Heidelberg, Germany

* These authors contributed equally to this work

§ These authors jointly supervised this work

Corresponding authors

Florian Leuschner

Email: Florian.Leuschner@med.uni-heidelberg.de

Christina Armstrong
Email: armstrong.ccs@gmail.com

Address: Im Neuenheimer Feld 410, 69120 Heidelberg, Germany

Telephone number: +49 6221 56 7317

**Table S1: Patients rejected from statistical analysis due to not available valve culture**

| **n** | **Blood culture** | **Valve PCR** | **Valve culture** |
| --- | --- | --- | --- |
| 1 | *Staphylococcus epidermidis* | *-* | n/a |
| 2 | *Staphylococcus epidermidis* | *Staphylococcus aureus* | n/a |
| 3 | *Staphylococcus aureus* | *Staphylococcus aureus* | n/a |
| 4 | *Corynebacterium jeikeium* | *Tropheryma whipplei* | n/a |

−: Negative; n/a: not available results

**List 1: Other microorganisms detected**

**n=27 other microorganisms detected by all methods included:**

1 *Achromobacter spp.*, 1 *Aggregatibacter actinomycetemcomitans*, 2 *Candida spp.*, 4 *Cardiobacterium hominis*, 1 *Clostridium perfringens*, 3 *Corynebacterium spp.*, 2 *Coxiella burnetii*, 1 *Escherichia coli*, 1 *Klebsiella oxytoca*, 2 *Neisseria elongata*, 1 *Paracoccus yeei*, 2 *Propionibacterium acnes,* 1 *Pseudomonas aeruginosa*, 1 *Rothia mucilaginosa*, 2 *Tropheryma whipplei,* and 1 eubacterial DNA, which could not be further specified.

**n=11 other microorganisms detected by blood culture:**

3 *Cardiobacterium hominis*, 1 *Clostridium perfringens*, 1 *Corynebacterium* spp*.*, 1 *Escherichia coli*, 2 *Neisseria elongata*, 1 *Propionibacterium acnes,* 1 *Pseudomonas aeruginosa*, and 1 *Rothia mucilaginosa*.

**n=14 other microorganisms detected by valve PCR:**

1 *Aggregatibacter actinomycetemcomitans, 1* *Cardiobacterium hominis*, 2 *Corynebacterium spp.*, 2 *Coxiella burnetii*, 1 *Klebsiella oxytoca*, 1 *Neisseria elongata*, 1 *Paracoccus yeei*, 1 *Propionibacterium acnes,* 1 *Rothia mucilaginosa*, 2 *Tropheryma whipplei* and 1 eubacterial DNA, which could not be further specified.

**n=7 other microorganisms detected by valve culture:**

1 *Achromobacter spp.*, 2 *Candida spp*., 1 *Enterobacter cloacae*, 1 *Rothia mucilaginosa*, and *2* *Propionibacterium acnes.*

**Table S2: Patients with multiple detections, 7 in blood and 4 in valve cultures**

| **n** | **Blood culture** | **Valve PCR** | **Valve culture** |
| --- | --- | --- | --- |
| 1 | *Enterococcus faecalis, Staphylococcus haemolyticus* | *Enterococcus faecalis* | *-* |
| 2 | *Staphylococcus capitis/epidermidis, Cardiobacterium hominis* | *Staphylococcus capitis/epidermidis* | *-* |
| 3 | *Streptococcus mitis/oralis, Staphylococcus spp.* | *Streptococcus oralis* | *-* |
| 4 | *Staphylococcus aureus, Propionibacterium acnes* | *-* | *-* |
| 5 | *Staphylococcus aureus, Staphylococcus epidermidis* | *-* | *-* |
| 6 | *Cardiobacterium hominis, Enterococcus faecalis* | *Enterococcus faecalis* | *-* |
| 7 | *Clostridium perfringens, Streptococcus sanguinis* | *Streptococcus sanguinis* | *-* |
| 8 | *Streptococcus gordonii* | *Streptococcus gordonii/oralis* | *Achromobacter spp., Streptococcus gordonii* |
| 9 | n/a | *-* | *Propionibacterium acnes, Staphylococcus pettenkoferi* |
| 10 | *-* | *Staphylococcus aureus* | *Staphylococcus aureus, Enterobacter cloacae* |
| 11 | *-* | *Propionibacterium acnes* | *Staphylococcus epidermidis, Propionibacterium acnes* |

−: Negative; n/a: not available results

**Table S3: Patients with different species between methods**

| **n** | **Blood culture** | **Valve PCR** | **Valve culture** |
| --- | --- | --- | --- |
| 1 | *Corynebacterium jeikeium* | *Tropheryma whipplei* | n/a |
| 2 | *Staphylococcus aureus* | *Corynebacterium tuberculostearicum* | - |
| 3 | *Staphylococcus epidermidis* | *Staphylococcus aureus* | n/a |
| 4 | *Staphylococcus epidermidis* | *Coxiella burnetii* | *-* |
| 5 | *Staphylococcus spp.* | *Streptococcus mitis/oralis* | *-* |
| 6 | *Neisseria elongata* | *Paracoccus yeei* | *-* |
| 7 | *Staphylococcus aureus* | *-* | *Candida parapsilosis* |
| 8 | *Staphylococcus aureus* | *Staphylococcus aureus* | *Candida guilliemondi* |
| 9 | *Staphylococcus aureus* | *Staphylococcus haemolyticus* | *Staphylococcus haemolyticus* |

−: Negative; n/a: not available results
